# Before the Lords' Committee

**Published:** 1890-06-07

**Authors:** 


### Before the Lords' Committee.

(By Our Special Commissioner.)

II.—SOME INTERESTING FACTS.

Dr. Steele has been the medical superintendent of Guy's Hospital for thirty-five years, and during the six previous years he was medical superintendent of the Glasgow Royal Infirmary. Dr. Steele has thus had forty-one years' continuous experience of hospital administration, and is, therefore, the oldest hospital official in the country with the exception of Mr. Wm. J. Nixon, of the London, whose experience extends to upwards of forty-four years. It is satisfactory to note that Dr. Steele is able to declare emphatically that the system of management at the voluntary hospitals throughout the British Empire is very much improved in every particular, including diet, nursing, and medical attendance. It appears that each senior physician and senior surgeon at Guy's has on an average forty-five beds, whilst the assistant physicians and surgeons have not more than six or eight each. At many of the hospitals, however, the latter have no beds at all. The seniors visit the hospital three times a-week as a rule, the assistants being much oftener in attendance. There are eight house physicians and house surgeons resident in the hospital, in addition to more than one hundred medical students employed in the wards in various capacities. Two resident obstetricians and from six to eight extern clerks attend about three thousand women annually in the maternity department. Sometimes as many as a dozen confinements are attended within twenty-four hours. There are six dispensers engaged, a senior and five juniors, the former receiving a salary of £180, the latter being paid from £70 upwards. Apart from the matron's department, the only paid residents at Guy's are the medical superintendent and the chaplain. The senior physicians and senior surgeons receive an honorarium of £40 a-year each, and the assistant physicians and surgeons a retaining fee of £100 a-year each, but the aurist and dentist receive no pay. From forty to fifty lecturers and instructors are engaged in the medical school, the annual income of which amounts to from £10,000 to £12,000. These fees are placed in the common purse, and, after payment of the necessary expenses, are divided into so many shares, the Committee, who regulate the distribution, apportioning a larger amount to the seniors than the juniors; but he referred the Committee to the dean of the school for information on these points. Each student at a London Medical School pays from £100 to £140. The house surgeon and house physician on duty for the week practically decide whether or not an applicant shall be admitted to in-patient treatment. Dr. Steele is of opinion that the advice given at the general hospitals in London is the very best that can be got, and the same may be said of the treatment at the Hospital for Consumption at Brompton and the National Hospital for the Paralysed and Epileptic in Queen Square. About one hundred patients are admitted on an average to Guy's every week. Incidentally, Dr. Steele stated that cases of diphtheria are still admitted to the general wards of Guy's Hospital. Experience has shown, however, that this practice ought to be discontinued in the interests of the other patients, especially now that the Metropolitan Asylums Board have made special provision in their hospitals for isolating this class of infectious disease.

Special Hospitals.

Dr. Steele is of opinion that special hospitals for consumption, for diseases of the chest, for eye diseases, for lying-in cases, and for children are a necessity, but now that the general hospitals have special departments for diseases of particular organs of the body other special hospitals are not needed. He is, therefore, opposed to orthopædic hospitals, to hospitals for diseases of the throat, the skin, &c., as neither necessary nor desirable. Dr. Steele objects to special hospitals because of the great difficulty placed by them in the way of patients receiving admission, owing to the institution of a registration fee. This is remarkable seeing that a registration fee of threepence is charged to every out-patient at Guy's Hospital who can afford it, and for the further fact that all, or nearly all, the special hospitals admit very many cases without exacting any registration fee, or indeed any payment whatever. It seems to us that Dr. Steele's evidence on this point is not quite consistent owing no doubt to a misapprehension on his part of the system in force at the special hospitals.

The Government of Guy's Hospital.

There are sixty governors at Guy's, the large majority of whom are Conservatives. Mr. W. E. Gladstone, the oldest governor, was appointed in 1833, because he was supposed at that time to be the rising hope of the Conservative Party. The governors are cooptative. There is no monetary qualification, and the governors themselves fill up any vacancy at their will and pleasure. They have very little to do in connection with the management of the hospital, being specially charged with the duty of caring for the hospital property, the estates, and any changes which may occur in relation thereto. The endowed income at the present time is about £25,000 per annum. The treasurer is the responsible officer who really manages the hospital, and the estates with the assistance of land stewards, the governors merely hearing the reports of their agents and never interfering with the internal management of the hospital at all. The Court of Governors meet every six weeks, seven being a quorum. There is no building or finance committee, but a chartered accountant acts as auditor, the books being kept by a paid accountant appointed by the treasurer. If the auditor disapproves of any matter connected with the accounts it is his duty to report to the treasurer, who need not bring the matter to the notice of the governors unless he chooses to do so. Dr. Steele is strongly of opinion that the treasurer's hands would be much strengthened if there was a weekly Board or Committee in connection with Guy's Hospital. There is a medical and nursing committee, consisting of two members of the medical staff and four or five governors, which meets every month. The members of the monthly committee are appointed by the governors and the two medical members by the medical staff. There is a separate medical committee made up of the medical officers attached to the hospital.

### Nursing Department.

The Matron is the responsible head of the nurses, but she cannot select nurses or do anything with them without the sanction of the Medical Superintendent, and the approval of the Treasurer. The nursing staff consists of Sisters or head nurses, of whom there are twenty; staff nurses and probationers, of whom there are about one hundred and ten. The nursing of a ward of twenty-two patients is managed by one Sister, one staff nurse, one probationer, and one lady pupil, in addition to a night nurse who is on duty all night from ten p.m. to eight a.m. No nurses are engaged under twenty-three years of age, and no lady pupil under twenty-four years of age. The latter pay for their board and lodging, uniform, and washing. A probationer receives £1 a month for the first year, in addition to uniform, board, lodging, and washing. For the next two years she receives £18 a year in wages, and at the end of three years is entitled to a certificate of training, when her wages are increased at the rate of £2 a year up to £36 per annum. Lady pupils remain for one year, during which they are taught the practice of medical and surgical nursing. Many lady probationers at the end of a year or two become Sisters in charge of wards. The hospital is affiliated to the National Pension Fund for Nurses, and the governors undertake to pay one-half of the premiums of all the nurses who join the fund so long as they remain in the service of the hospital. The Nurses' Institute attached to the hospital supplies private nurses to the public, fifty nurses being employed, and the hospital receives a considerable revenue from this source. The charge to the public for a nurse varies from a guinea and a half to two guineas a week. Experience shows that a nurse has to get acclimatised to a hospital, and although cases of hospital sore throat are now comparatively rare, it takes a nurse from three to four months before she gets regularly habituated to hospital work.

### Out-patients.

Out-patients are seen every day at Guy's Hospital; medical cases on three days, surgical cases on four days a week; and special complaints, such as skin, throat, eye, teeth, and diseases peculiar to women on other days. Each out-patient has to pay threepence a week for medicine. Dr. Steele's opinion is that the patients have readily fallen in with the plan and that the same class of people as formerly still attend in the out-patient department. At first the number of applicants in the out-patient department diminished, but latterly the numbers have so increased that the authorities have been compelled to limit the numbers, and it frequently happens that twenty people are sent away without treatment. Out-patients may be kept waiting a considerable time (four hours), before they see the doctors, but there is a refreshment bar in the out-patient department, and Dr. Steele says that it would be impossible to obviate these delays. Four members of the medical staff see out-patients every day. Two senior students each day first examine all new cases and distribute eighty cards (*i.e.*, forty medical and forty surgical), subject to the control of the house physician or house surgeon on duty. When the eighty cards have been distributed the remainder of the patients are sent away, but it is the duty of the house physician to see that none of them are very ill, and should he discover any particular symptom he has the power to issue further cards to all such cases so as to prevent anyone being sent away who is at all seriously ill. Altogether about four or five hundred out-patients are seen on one day at Guy's, *i.e.*, each member of the staff on duty attends to 125 patients, whom he sees at the rate of thirty per hour, so that he is enabled to give two minutes on an average to each patient. Dr. Steele is of opinion that it is not the very poor who apply for relief in the out-patient department, but the lower middleclass, trades-people, and members of the working classes. The very poor go to the Poor-Law dispensaries. Dr. Steele stated that there were twelve thousand beds in the Poor-Law infirmaries in London and only four thousand in special and general hospitals which are occupied on an average. This is clearly an error seeing that there are upwards of eight thousand beds in the general and special hospitals, of which about six thousand are constantly occupied on an average. Dr. Steele is of opinion that the out-patient cases are those which the students are likely to meet with in practice afterwards and are thus very useful for purposes of instruction. He further holds that the inconvenience attending the crowded condition of the out-patient departments of the hospitals must be put up with by the patients.

### Poor-law Infirmaries and Clinical Instruction.

There is a general feeling amongst the teachers in the medical profession that Poor-Law infirmaries should be open under certain restrictions to medical students and also to the surgeons and physicians attending the large hospitals. Dr. Steele rather favours the view that the guardians ought to enter into an arrangement with the authorities of the general hospitals to transfer from the infirmaries all cases of clinical interest that would be instructive to students. The plan adopted by a few of the Poor-Law infirmaries of allowing the medical schools to send one or two students to take pathological notes and to make post-mortem examinations ought to be extended to all those institutions. Arrangements should be made for the attendance of visiting surgeons and visiting physicians and a class of students to see the cases in the infirmaries. We think Dr. Steele is wrong, however, in holding that Poor-Law infirmaries would not be very attractive to the mass of the medical students in London owing to their distance from the clinical hospitals. Besides, there are many eminent and able members of the profession apart from those actively engaged at the medical schools who would gladly attach themselves to Poor-Law infirmaries if they had the opportunity. Such an arrangement could not fail to increase the efficiency of the medical treatment, and must in many ways tend to confer material benefit upon the patients.

### A Suggestion for the Committee.

It is very difficult to follow the evidence given by each witness under the existing circumstances. The same question is taken up over and over again and so avoidable difficulties are caused. We would suggest that each witness should be asked to forward to the Chairman the heads of the evidence he proposes to give. These heads, together with any points the Committee attach importance to might then be classified, and a copy of them given at the commencement of the sitting to each member as well as to the witness. The examination-in-chief could then proceed in regular order, and so soon as the witness had completed his evidence-in-chief on a given point, members of the Committee could put their questions to him and so each section of the evidence would be altogether in a complete form. This would effect a great saving of time and add much to the value and clearness of the facts elicited, besides simplifying the work of the Committee itself.

